# Development and characterization of lactoferrin nanoliposome: cellular uptake and stability

**DOI:** 10.1186/1556-276X-7-679

**Published:** 2012-12-17

**Authors:** Rongfa Guan, Jieqing Ma, Yihang Wu, Fei Lu, Chaogeng Xiao, Han Jiang, Tianshu Kang

**Affiliations:** 1 Zhejiang Provincial Engineering Laboratory of Quality Controlling Technology and Instrumentation for Marine Food, China Jiliang University, Hangzhou 310018, People’s Republic of China; 2 Zhejiang University of Technology, Hangzhou 310014, People’s Republic of China; 3 Food Science Institute, Zhejiang Academy of Agricultural Sciences, Hangzhou 310021, People’s Republic of China

**Keywords:** Lactoferrin, Nanoliposome, Cellular uptake, Stability

## Abstract

Lactoferrin was purported in consumer literature to enhance and support the immune system response through their antioxidant, antibacterial, and anticarcinogenic properties. To improve the effectiveness of lactoferrin, liposomes were used as a carrier in this study. The main purpose of this study was to compare three different methods to prepare the lactoferrin nanoliposomes based on the encapsulation efficiency and size distribution and evaluate the stability and cellular uptake of lactoferrin nanoliposomes. Encapsulation efficiency and size distribution indicated the reverse-phase evaporation method was fit for preparing the lactoferrin nanoliposomes. The stabilities of lactoferrin nanoliposomes in simulated gastrointestinal juice, sonication treatment time and lipoperoxidation extent of storage time were evaluated. The lactoferrin nanoliposomes showed an acceptable stability in simulated gastrointestinal juice at 37°C for 4 h and short treatment times were required to achieve nano-scaled liposomes. Furthermore, the viability of cells was decreased by increasing the concentration of the various lactoferrin nanoliposomes. The methyl thiazolyl tetrazolium results demonstrated that Lf nanoliposomes and Lf activated in the cells in a manner of dose-effect relation and Lf nanoliposomes had a statistically significantly different (p<0.01) between the concentration 5 and 10 mg/mL. According to the results, nanoliposomes may be fit for the oral administration of lactoferrin and could be useful approach for lactoferrin availability in tumor cells.

## Background

Lactoferrin (Lf) is an 80 kDa iron-binding glycoprotein of the transferrin family, which was first isolated from milk by Groves [1]. Lf is abundant in milk and other biological fluids, such as tears, saliva, mucous, pancreatic juice, bile and so on. Lf is a protein with multiple biological functions, and it is not only involved in iron transport, but also has immune response, tioxidant activities, antimicrobial activities, especially in anticarcinogenic activities [2-6]. Bezault found that Lf made solid tumor growth decreased and strongly inhibited experimental metastasis in mice [7]. In addition, Campbell [8] had demonstrated that Lf may be down-regulated in some cancers, such as human breast carcinoma and showed that it may regulate cell proliferation.

As a vital role in food, proteins were able to form gels and emulsions, which allowed them to be an important material for the encapsulation of bioactive compounds [9,10].

One of the significant efforts towards this aim had been the use of colloidal delivery systems such as liposomes, micro- or nanoparticles [11]. There had been considerable interest in liposomes [12], as they may be used for protection in food and pharmacy system [13-16]. Besides, nanoliposomes had the advantages of nanoparticles, which improved the targeting and absorption into the intestinal epithelial cells [17,18]. In this case, nanoliposomes could be used as a potential carrier in food system.

The aim of present study was to choose the best method to develop the Lf nanoliposomes and investigate the stability of Lf nanoliposomes under different conditions, especially in the simulated gastrointestinal tract. The nanoliposomes were characterized by means of encapsulation efficiency and particle size. Furthermore, the Lf nanoliposomes were investigated to evaluate the cellular uptake and the effect on tumor cells.

## Material and methods

### Materials

Phosphatidylcholine (PC) was purchased from Beijing Shuangxuan Microbe Culture Medium Products Factory (Beijing, China). Cholesterol (CH), pepsin and steapsin were obtained from Shanghai Chemical Reagent Co. (Shanghai, China). Lactoferrin was purchased from Seebio Company (Shanghai, China). Chloroform, diethyl ether and Tween 80 were obtained from Hangzhou Jiachen Chemical Company. All chemicals were of reagent grade and used without further purification.

### Methods

#### Lactoferrin nanoliposomes preparation

Three different methods were carried out to prepare Lf nanoliposomes.

#### Reverse-phase evaporation method

Lf nanoliposomes were prepared by reverse-phase evaporation method [19]. Briefly, a certain amount of PC and CH were dissolved in chloroform-diethyl ether and Lf was dissolved in phosphate buffer solution (pH7.4). The organic phase was mixed with the aqueous phase using probe sonication for 5 min. The mixture was placed in a round-bottom flask and a gel was formed by evaporating the organic solvent under reduced pressure at 35°C using a rotary evaporator. Then 30 mL phosphate buffer solution (0.20 M, pH 7.4, PBS) containing tween 80 was added and evaporated for another 20 min.

#### Film method

The method of preparing Lf nanoliposomes was described by Bangham and Lea [20]. Lipids were dissolved in chloroform-diethyl ether forming a mixture. The organic solvent was then removed by rotary evaporation under reduced pressure at 35°C using a rotary evaporator. The dry lipid film was hydrated with a solution of Lf dissolved in phosphate buffer solution (0.20 M, pH 7.4, PBS).

#### Ether injection method

The method of preparing Lf liposomes was described by Dream and Bangham [21]. PC and Chol were dissolved in a certain volume of diethyl ether and the Lf was dissolved in amount of phosphate buffer (0.02 M, pH7.4). The organic solution was injected into the aqueous solution. The mixture was placed into a glass bottle fitted with a silicone rubber injection cap and this bottle was placed in a water jacket connected to a circulating water bath maintained at 35°C with rapid mixing until diethyl ether removed.

#### Characterization of lactoferrin liposomes

The particle size was measured by Mastersizer 2000 instrument (Malvern), equipped with HydroMu dispersing unit (Malvern). Measurements were taken in the range between 0.1 and 1000 μm, under the following conditions: water refractive index 1.33, and general calculation model for irregular particles. The data obtained were averaged by software (Mastersizer 2000).

#### Encapsulation efficiency determination (EE)

The encapsulation efficiency was determined by centrifuge-UV method. Take nanolipsomes suspension (500 μL) by spinning at 10000 rpm for 30 min using centrifuge, the protein content of the supernatant was measured by Bradford. The same suspension was ruptured using sufficient volume of ethanol, and the total amount of Lf was determined spectrophotometrically.

Encapsulation efficiency was calculated using Eq.1.

(1) $$\text{EE}\%=\frac{Q_{t}-Q_{f}}{Qt}*100$$

Where Q_f_ is the amount of free Lf and Qt is the total amount of Lf present in 500 μL of nanoliposomes.

### Stability of lactoferrin liposome

#### Malondialdehyde (MDA) Value

Lf nanoliposomes were stored in a refrigeratory at 4°C. The MDA value was determined as an index of the PL peroxidation. The MDA value was detected spectrophotometrtically by the thiobarbituric acid (TBA) reaction following the method of Weng and Chen [22]. Taking 5 mL of a mixture of 25 mmol/L TBA, 0.9 mol/L TCA and 50 mmol/L HC1 in a test-tube and 1 mL Lf nanoliposomes was heated to 100°C for 30 min and After reaching the room temperature, the absorbance of the solutions were measured at 535 nm.

#### Effect of sonication

Lf nanoliposomes (10 ml) were put into a 30 mL beaker and were ultrasonicated with a probe sonicator (VCX400, Sonics & Material, Inc., USA) in an ice bath with 1 s ON, 1 s OFF intervals. Samples of 0.2 mL were taken at predetermine intervals. Encapsulation efficiency of withdrawn samples was determined. The release ratios were calculated.

#### In vitro release of lactoferrin from nanoliposomes

The controlled release was examined in simulated gastric juice of pH 1.3 and intestinal juice of pH 7.5. The solution of pH 1.3 consisted of HCl(0.10 M), pepsin and deionized water, while the solution of pH 7.5 was made up of KH_2_PO_4_ (6.8 mg/mL), NaOH (0.10 M, adjusted to pH 7.5), Trypsin (10 mg/mL) and deionized water. Five milliliters of Lf nanoliposome suspension was mixed with the equal volume of simulated gastrointestinal juice in a 50 mL beaker. The beaker was placed on a magnetic stirrer adjusted to a constant speed of 150 rpm at 37°C. Aliquots of 0.2 mL were sampled from the beaker at predetermine intervals. The release of Lf from nanoliposomes was evaluated by release ratio. The release ratio was calculated using the Eq. (2).

(2) $$\text{Release ratio \%}=\left(1-\frac{{\text{EE}}_{t}}{{\text{EE}}_{0}}\right)\times 100$$

Where EE_0_ is the encapsulation efficiency of lactoferrin nano-liposomes before incubation and EE_t_ is the encapsulation efficiency of lactoferrin nanoliposomes after incubation for the time.

### Cellular uptake studies

Cell viability was measured by the MTT assay [23]. Caco-2 cells (CBCAS, Shanghai, China) were cultured in DMEM (Gibco, MD, US). Cells were cultured at 37°C with 5% CO_2_. Cells were passaged thrice a week. At 80% confluence, the cells were subcultured into the 96-well plates. After the monolayer of cells became formed for 36 h, cells were treated with a range of concentrations of different Lf nanoliposomes and Lf. After the 24 h treatment, we renewed the serum-free medium containing 3-(4, 5-dimethylthiazol-2-yl)-2, 5-diphenyltetrazolium bromide (MTT, 0.5 mg.ml^-1^) and allowed to grow for another 4 h. The MTT assay assessed cell-viability by measuring the enzymatic reduction of yellow tetrazolium MTT to a purple formazan, as measured at 570 nm using Enzyme-labeled instrument (Tecan Co.) [24,25].

### Statistical analysis

The results were expressed as the mean ± standard deviation (SD). The statistical study was performed using SPSS, version 15.0 for windows.

## Results and discussion

### Characterization of lactoferrin liposomes

#### Particle size

**Figure 1:**
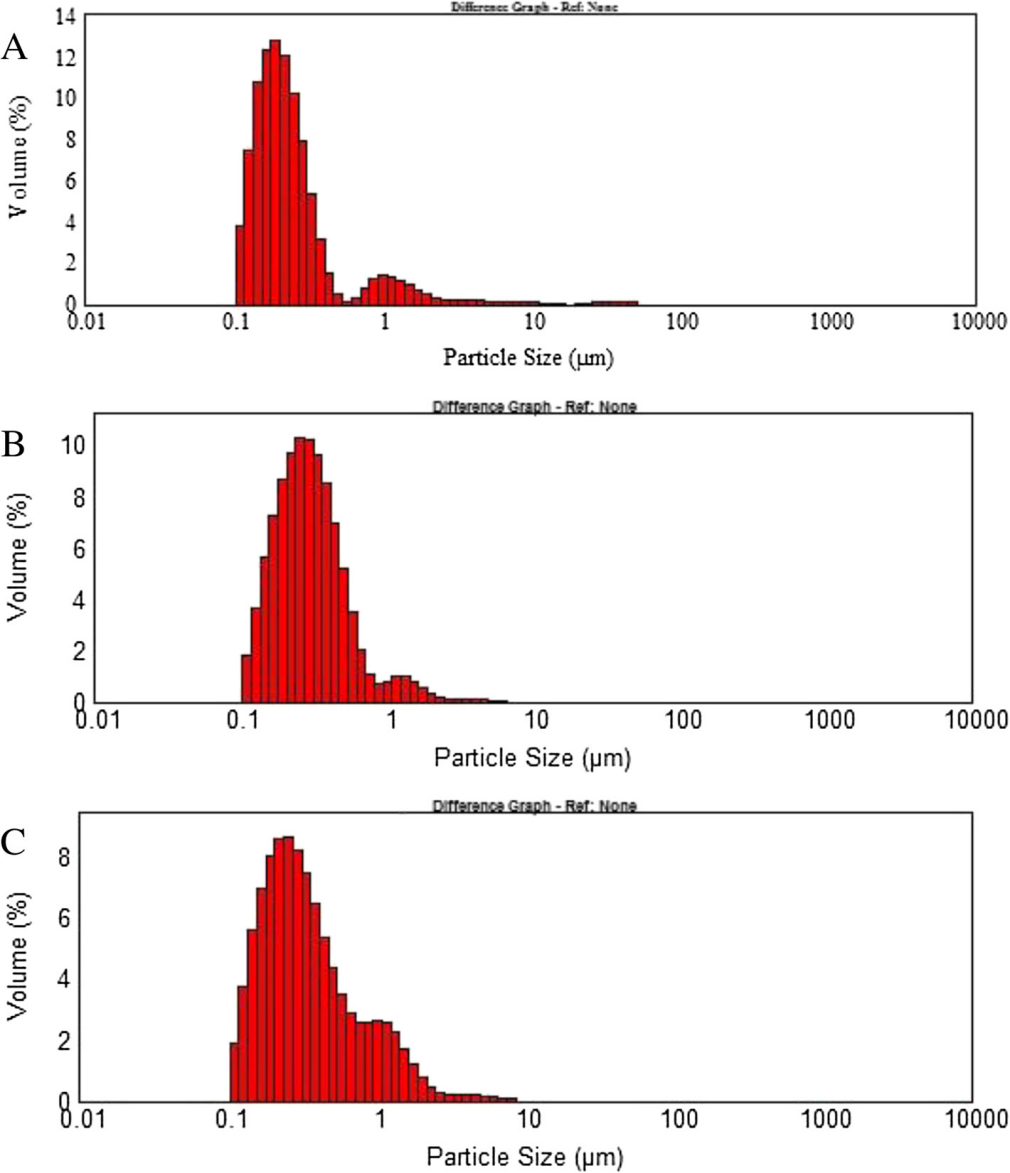
Size distribution of Lf liposomes prepared by (A) reverse-phase evaporation method, (B) ether injection method (C) film method, after 20 min of sonication at ice bath and pH 7.4.

In Figure 1 it can be observed size distribution of Lf nanoliposomes prepared by the three different methods. The size distribution is generally used as a characterization tool to evaluate the stability of Lf nanoliposomes. The result showed that the size of nanoliposomes was ranked in the following order, reverse-phase evaporation method< ether injection method <film method.

#### Encapsulation efficiency

**Figure 2:**
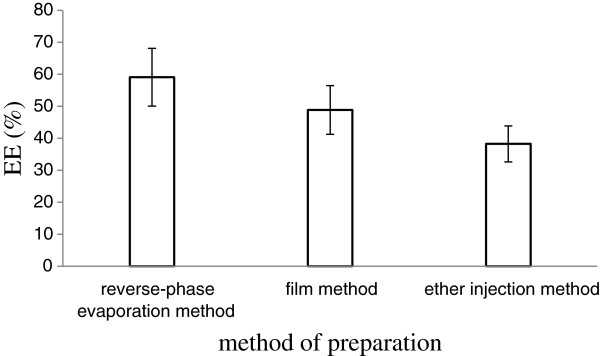
**The effect of three different methods on encapsulation efficiency of lactoferrin nanoliposomes.** Data reported are the mean values ± standard variation of three replications.

The effect of three different methods on encapsulation efficiency of Lf nanoliposomes is shown in Figure 2. The encapsulation efficiency prepared with reverse-phase evaporation method, ether injection method and film method was 50.1%, 34.5% and 48.9%, respectively.

Above all, reverse-phase evaporation method is a simple and applicable operation to most of the phospholipid mixture encapsulation volume and has high encapsulation efficiency. This method is suitable for wrapping water soluble drugs and macromolecular biologically active substance.

### Stability of lactoferrin liposome

#### Malondialdehyde (MDA) value

**Figure 3:**
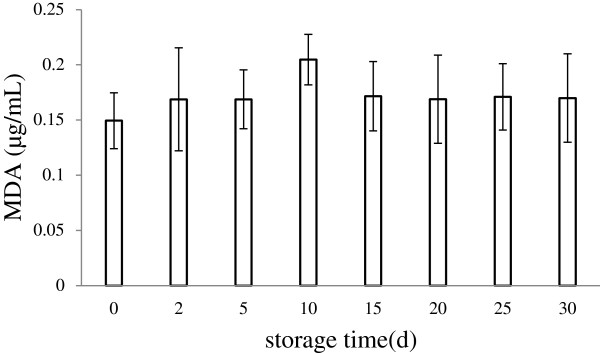
**Variation of the MDA values in Lf nanoliposomes during storage at 4°C for 30 days.** Data reported are the mean values ± standard variation of three replications.

Phospholipid was used as the major component of liposomal membrane, containing partially polyunsaturated fatty acid residues sensitive to oxidative free radicles [26]. The MDA, which as a final product of fatty acid peroxidation was evaluated in the study. During 30 days of storage at 4°C, the MDA values in Lf nanoliposomes showed no distinct differences in the MDA values were shown in Figure 3. The result showed the Lf nanoliposomes could be stable in a period of time.

#### Effect of sonication

**Figure 4:**
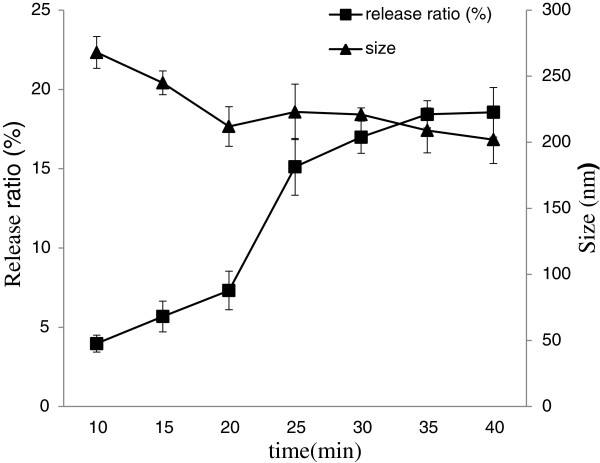
**The effect of particle size and release ratio on Lf nanoliposomes after storage at 4°C.** Data reported are the mean values ± standard variation of three replications.

Sonication was used to form a w/o emulsion with the REV method, and to control and reduce the size of microvesicles [27]. The stability of Lf nanoliposomes was evaluated by measuring the change of particle size and release ratio after storage at 4°C for 30 days was shown in Figure 4. After 25 min sonication on nanoliposomes, the release ratio was 15.12% which was higher than the release ratio of 20 min sonication. This may be caused by the extension of ultrasonic total time, the crushed particles had been gained new energy, resulting in a change of stability . With time increased, the size of nanoliposomes became smaller. This is due to ultrasound phenomena in liquid media enhance mass transports of their constituents in a non-homogeneous fashion allowing the fast formation of vesicles [28]. After 20 min sonication on Lf nanoliposomes, the particle size did not change so much. Above all, the 20 min sonication time on Lf nanoliposomes may be fit for preparing.

#### In vitro release of lactoferrin from nanoliposomes

When Lf nanoliposomes could be used as carriers for the oral administration of Lf, they must be able to withstand passage through the stomach and small intestine. In vitro release has been used as a very important surrogate indicator of in vivo performance.

**Figure 5:**
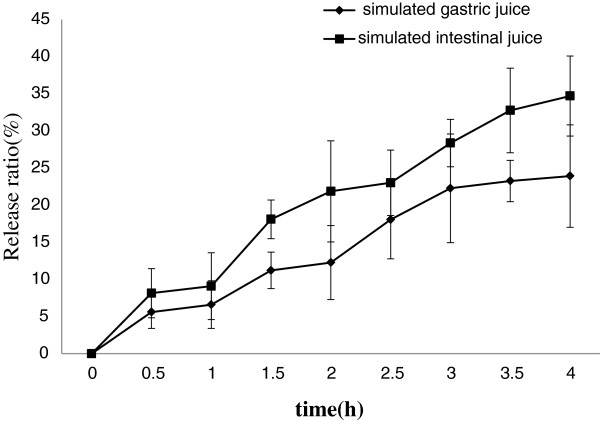
**The effect of simulated gastrointestinal juice on nanoliposomes.** Data reported are the mean values ± standard variation of three replications.

Figure 5 showed the release ratio of Lf from nanoliposomes in simulated gastrointestinal juice. About 23% Lf was released from nanoliposomes within 4 h in the simulated gastric juice. However, because food usually remains in the stomach for more or less 4 h, the liposomal Lf could be effectively protected in the gastric juice [29]. In simulated intestinal juice, bile salts and pancreatic lipase may cause the Lf release from nanoliposomes [30]. This phenomenon may increase the release of nanoliposome.

#### Cell viability

**Figure 6:**
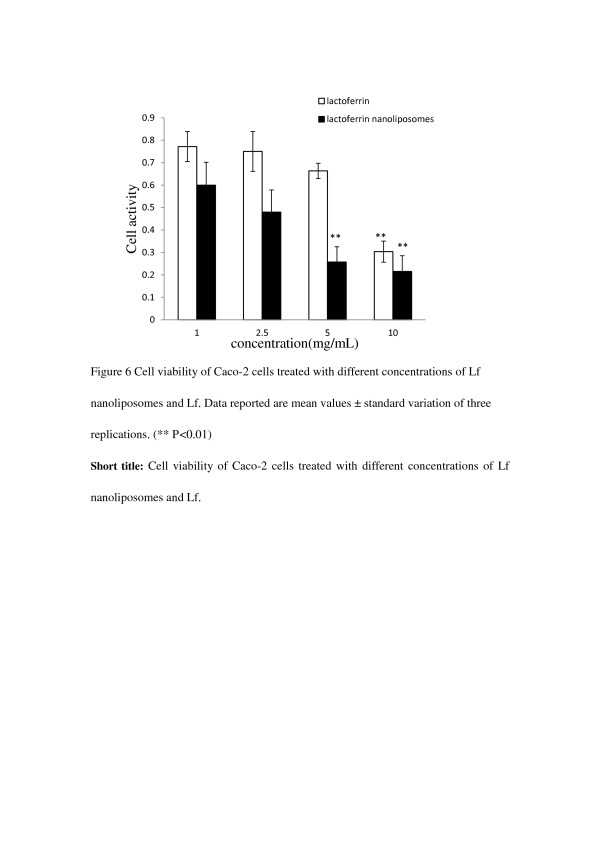
**Cell viability of Caco-2 cells treated with different concentrations of nanoliposomes and Lf.** Data reported are the mean values ± standard variation of three replications.(** P<0.01).

After cells were incubated with 1, 2.5, 5, 10 mg·mL^-1^ of Lf nanoliposomes for 24 h, compared with the Lf in control experiments. Figure 6 showed the MTT results demonstrated a concentration dependent uptake after exposure to Lf nanoliposomes. With the same concentration, the cell activity of Lf nanoliposomes is lower than the cell activity of Lf. It is observed that the cell activity is a statistically significantly different (p<0.01) between the concentration 5 and 10 mg/mL. The MTT results showed that Lf nanoliposomes and Lf activated in the cells in a manner of dose-effect relation and Lf nanoliposomes has a more obvious function to the cells (*p*<0.01). The possibility of both targeting drugs to specific tissues and cells, and facilitating their uptake and cytoplasmic delivery has rendered liposomes a versatile drug carrier system with numerous potential applications in medicine [31].

## Conclusions

Lf nanoliposomes with high encapsulation efficiency were prepared successfully by REV method. The particle size indicated the stability of the Lf nanoliposomes suspension. Lf nanoliposomes were tested in vitro for their stability in simulated gastrointestinal juice. The Lf nanoliposomes showed an acceptable stability in simulated gastrointestinal juice at 37°C for 4 h. According to the results, Lf nanoliposomes may be fit for use in the oral administration. The uptake of Lf nanoliposomes formulations were found to depend on concentration. In conclusion, we have demonstrated that Lf nanoliposomes with different concentration could modulate the growth of tumor cells.

## Abbreviations

Lf: Lactoferrin; MDA: Malondialdehyde; MTT: Methyl thiazolyl tetrazolium.

## Competing interest

The authors declare that they have no competing interests.

## Authors’ contributions

JM conducted most of experiments that the manuscript mentioned and drafted the manuscript; RG came up with the idea, contributed to the design of the experiment and agreed with the paper’s publication. FL and YW analyzed the data and drew the pictures. CX, HJ and TK revised manuscript critically and make a few changes. All authors read and approved the final manuscript.
